# Retraction: A robust technique based on VLM and Frangi filter for retinal vessel extraction and denoising

**DOI:** 10.1371/journal.pone.0203418

**Published:** 2018-08-29

**Authors:** The PLOS ONE Editors

This article [1] was submitted September 27, 2016 and published February 12, 2018. After publication, it was brought to our attention that there is substantial overlap with the following article that was previously published in the journal *IET Image Processing*:

“Robust Retinal Vessel Segmentation using Vessel's Location Map and Frangi Enhancement Filter”

Authors: Muhammad Shahid, Imtiaz Ahmad Taj [2]

Submitted: May 4, 2017; Published: December 6, 2017

Having confirmed the overlap, the *PLOS ONE* Editors retract this article, as we consider that this constitutes redundant publication.

The authors have indicated that KBK, AAK, and AJ were not aware of the submission of the article to *IET Image Processing*, which occurred while the submission to *PLOS ONE* was under revision.

KBK, AAK, AJ, and MS agreed with the retraction.
